# Restless Legs Syndrome and Parkinson Disease: A Causal Relationship Between the Two Disorders?

**DOI:** 10.3389/fneur.2018.00551

**Published:** 2018-07-24

**Authors:** Luigi Ferini-Strambi, Giulia Carli, Francesca Casoni, Andrea Galbiati

**Affiliations:** ^1^Department of Clinical Neurosciences, IRCCS San Raffaele Scientific Institute, Neurology – Sleep Disorders Center, Milan, Italy; ^2^Università Vita-Salute San Raffaele, Milan, Italy

**Keywords:** RLS/WED, PD, dopamine, iron, dopamine agonists

## Abstract

Restless Legs Syndrome/Willis-Ekbom Disease (RLS/WED) is a common sleep related movement disorder that can be idiopathic or occurs in comorbidity with other medical conditions such as polyneuropathy, iron deficiency anemia, multiple sclerosis, hypertension and cardiovascular diseases. In recent years, a growing body of literature investigated the association between RLS/WED and Parkinson's Disease (PD). Several questions regarding the comorbidity between these two disorders are still unanswered. If the insurgence of RLS/WED may precede the onset of PD, or if RLS/WED could represent a secondary condition of PD and if impaired dopaminergic pathway may represent a bridge between these two conditions are still debatable issues. In this review, we critically discuss the relationship between RLS/WED and PD by reviewing cross sectional and longitudinal studies, as well as the role of dopamine in these disorders. A twofold interpretation have to be taken into account: dopaminergic therapy may have a crucial role in the development of RLS/WED in PD patients or RLS/WED can be conceived as an early manifestation of PD rather than a risk factor. Several studies showed a high prevalence of RLS/WED in PD patients and several findings related to dopaminergic and iron alterations in both disorders, however up to now it is difficult to find a point of agreement between studies. A greater number of systematic and strongly controlled longitudinal studies as well as basic pathophysiological investigations particularly in RLS/WED are needed to clarify this complex relationship.

## Introduction

Restless Legs Syndrome/Willis-Ekbom Disease (RLS/WED) is a common sleep related movement disorder characterized by an urge to move the limbs frequently accompanied by uncomfortable and unpleasant sensations that are difficult to describe. Patients define their symptoms as burning, twitching, or pain in their lower limbs. However, in the most severe cases the symptomatology can be perceived also in the upper limbs (1). Onset of symptoms is frequent during period of rest or inactivity and an exacerbation of unpleasant sensations is reported in those situations where immobility is forced such as driving, flying long distance, watching movies in theater, and attending business meetings. Movement and motor activity typically relieved symptoms and patients may employ different strategies to alleviate the discomfort (2). RLS/WED has a clear circadian trend with a peak in the evening or at the night that can severely compromise nocturnal sleep quality and quantity. In accordance, patients commonly report insomnia symptoms characterized by difficulty to fall asleep or/and frequent nocturnal awakenings that disrupt sleep continuity. Therefore, daytime consequences such as irritability, fatigue, drowsiness, and cognitive impairments are usually reported (3). Remarkably, despite sleep macrostructure and microstructure is significantly altered by the presence of Periodic Limb Movements (PLM) sleepiness is not universally reported by these patients (4). In accordance, it has been argued that RLS/WED subjects may display a daytime hyperarousal state useful to compensate the negative effects of nocturnal impairments (5).

By employing minimal diagnostic criteria of the international restless legs syndrome study group (IRLSSG) the prevalence of this disorder has been estimated between 3.9% and 14.3% with women more affected than men and an increase with age (6). Notably, RLS/WED seems to have different prevalence linked to geographic areas: highest in European populations (5% to 12%), intermediate in Asian countries (1% to 8%), and lowest in African countries (<1%) (7).

RLS/WED is typically a chronic condition and requires a treatment in the long term. Different drugs have shown a good efficacy. In particular, dopamine agonists are effective in reducing patients' symptomatology and are considered first line treatment whereas Alpha-2-delta agonists are recognized as a valid alternative (8). However, after an initial amelioration, worsening, and re-emergence of symptoms are frequently reported. A well known iatrogenic side effect caused mostly by dopaminergic compounds is augmentation that can be defined as a worsening of symptomatology characterized by earlier onset of symptoms, shorter latency to symptom occurrence at rest, and spreading to other parts of the body. In this case the medication should be suspended or changed with an agent with minor probability of augmentation (9).

RLS/WED can be idiopathic but can also occur in comorbidity with other medical conditions. Genetic risk factors seem to be particularly related to the primary form of the disease, underlined by a high familiarity and the identification of some risk loci (10). Secondary form is described in several studies reporting its relationship with polyneuropathy (11), iron deficiency anemia (12), multiple sclerosis (13), hypertension, and cardiovascular diseases (14). However, in recent years a growing body of literature investigated the association between RLS/WED and Parkinson's Disease (PD). Several questions regarding the comorbidity between these two disorders are still unanswered. If the insurgence of RLS/WED may precede the onset of PD, or if RLS/WED could represent a secondary condition of PD and if impaired dopaminergic pathway may represent a bridge between these two conditions (15) are still debated topics. Jagota et al. (16) suggested that RLS/WED and PD may have similar impaired groups of neurons but a different pathophysiology. They argued that both types of patients respond to different therapies, except for the dopaminergic one. For example RLS/WED symptomatology improve with opioids and anticonvulsants while PD symptomatology has a good response to anticholinergic therapy. Thus, they supported the idea of an involvement of systems diverse than the dopaminergic one.

The aim of this paper is to critically discuss the relationship between RLS/WED and PD by reviewing cross-sectional and longitudinal studies, as well the role of dopamine in these disorders.

## Cross-sectional studies

**Figure 1:**
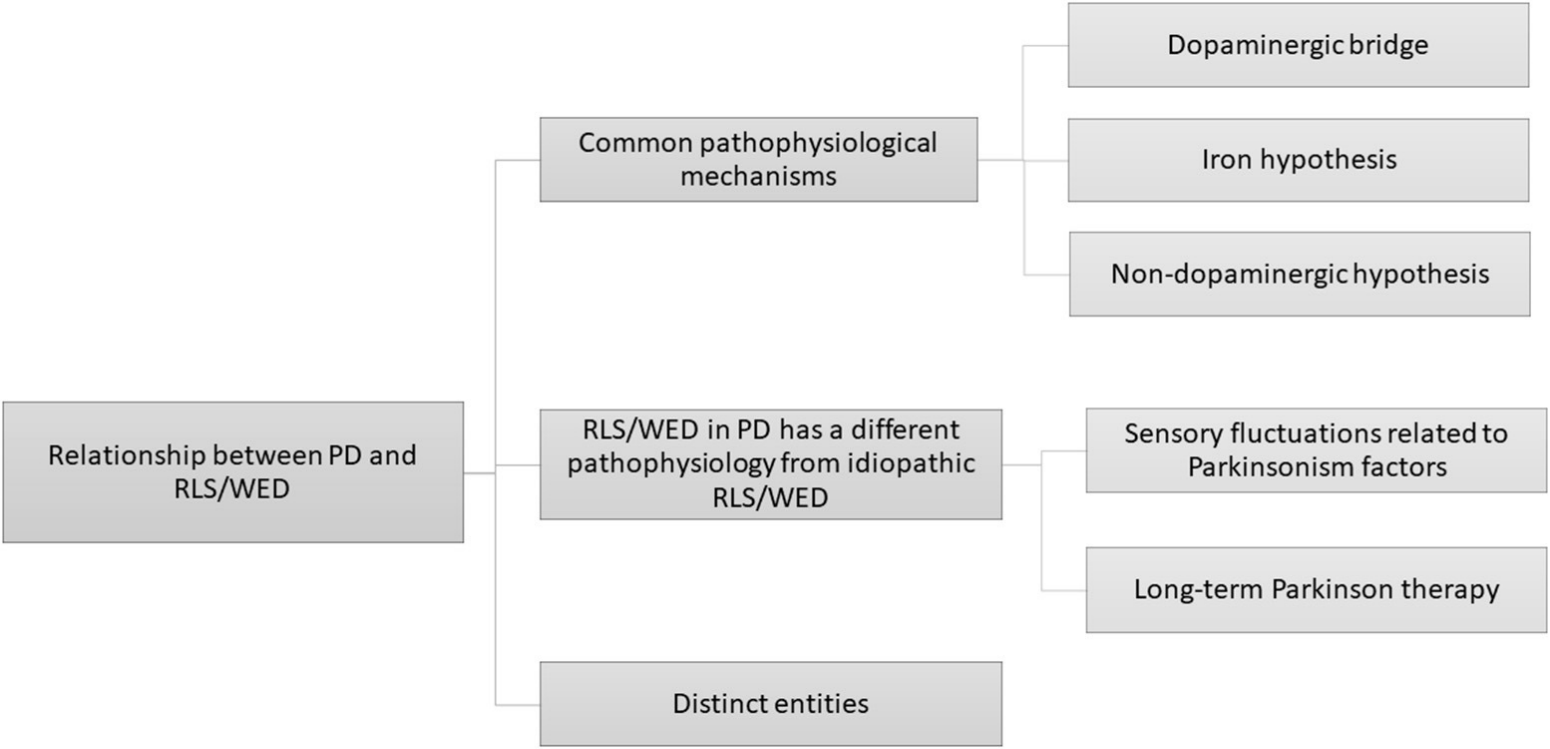
A graphical representation of several pathophysiological hypotheses proposed in literature regarding the relationship between PD and RLS/WED.

The literature regarding the prevalence of RLS/WED in PD patients presents conflictual findings leading to an open debate regarding this issue. Cross-sectional studies show a variable prevalence of RLS/WED in PD patients ranging approximately from 0 to 50% (Table 1). The evaluation of the prevalence of RLS/WED in PD patients can be useful to improve the knowledge of the relationship between these two diseases. RLS/WED and PD respond to dopaminergic therapy (8, 51, 52): this evidence suggest that the dopaminergic system may play a crucial role in both disorders. However, not all cross-sectional studies support this hypothesis (16, 20, 26, 27, 34, 36, 37, 45). The main hypotheses addressed by cross-sectional studies reported in this review are the following: (1) two diseases may share the same pathophysiological mechanism, (2) RLS/WED in PD has a different pathophysiology from the idiopathic RLS/WED (iRLS/WED) and (3) these two diseases are different entities (Figure 1).

**Table 1:** Cross-sectional study assessing the prevalence of RLS/WED in PD patients and general population.

**Article**	**Sample country**	**% RLS/WED in PD patients (n°)**	**% RLS/WED in controls (n°)**	**RLS/WED criteria**
(17)	USA	20.8 (63 out of 303)	N.A	IRLSSG diagnostic criteria of RLS/WED (1995) 18)
(19)	India	14.9 (21 out of 149)	1 (1 out of 115)	RLS/WED was referred to by a question
(20)	Singapore	0 (0 out of 125)	N.A	IRLSSG diagnostic criteria of RLS/WED (1995) 18)
(21)	India	7.9 (10 out of 126)	0.8 (1 out of 128)	IRLSSG diagnostic criteria of RLS/WED (2003) 22)
(23)	Brazil	52.3 (45 out of 86)	N.A	Irresistible desire to move the legs, particularly at night, aggravated by rest and ameliorated after movement.
(24)	Japan	12 (20 out of 165)	2.3 (3 out of 131)	IRLSSG diagnostic criteria of RLS/WED (2003) 22)
(25)	Spain	21.9 (25 out of 114)	N.A	IRLSSG diagnostic criteria of RLS/WED (2003) 22)
(26)	Singapore	3 (3 out of 200)	0.5 (1 out of 200)	IRLSSG diagnostic criteria of RLS/WED (2003) 22)
(27)	Italy	3.3 (4 out of 118)	2.7 (3 out of 110)	IRLSSG diagnostic criteria of RLS/WED (2003) 22)
(28)	Korea	16.3 (73 out of 447)	N.A	IRLSSG diagnostic criteria of RLS/WED (2003) 22)
(29)	France	0 (0 out of 11)	N.A	IRLSSG diagnostic criteria of RLS/WED (2003) 22)
(30)	Austria	24 (28 out of 113)	N.A	IRLSSG diagnostic criteria of RLS/WED (2003) 22)
(31)	Brazil	50 (8 out of 16)	0 (0 out of 12)	IRLSSG diagnostic criteria of RLS/WED (2003) 22)
(32)	Brazil	18.75 (9 out of 48)	N.A	IRLSSG diagnostic criteria of RLS/WED (1995) 18)
(34)	Netherlands	11 (29 out of 269)	N.A	IRLSSG diagnostic criteria of RLS/WED (2003) 22)
(16)	Thailand	1.6 (3 out of 183)	N.A	IRLSSG diagnostic criteria of RLS/WED (2003) 22)
(35)	Italy	2.75 (3 out of 109)	2.58 (3 out of 116)	IRLSSG diagnostic criteria of RLS/WED (2003) 22)
(36)	Norway	12.5 (21 out of 200)	6.9 (12 out of 173)	IRLSSG diagnostic criteria of RLS/WED (2003) 22)
(37)	Japan	5.5 (5 out of 93)	2.2 (2 out of 93)	IRLSSG diagnostic criteria of RLS/WED (2003) 22)
(38)	Norway	27 (47 out of 176)	N.A	IRLSSG diagnostic criteria of RLS/WED (2003) 22)
(39)	Malaysia	9.7 (11 out of 113)	N.A	IRLSSG diagnostic criteria of RLS/WED (2003) 22)
(40)	India	11.9 (16 out of 134)	2.9 (5 out of 172)	IRLSSG diagnostic criteria of RLS/WED (2003) 22)
(41)	Canada (diverse ethnic background)	21.3 (27 out of 127)	4.7 (6 out of 127)	RLS/WED diagnostic criteria (not specify)
(42)	UK	16.2 (6 out of 37)	10.8 (4 out of 37)	IRLSSG diagnostic criteria of RLS/WED (2003) 22)
(43)	South Korea	16.5 (25 out of 151)	N.A	IRLSSG diagnostic criteria of RLS/WED (2003) 22)
(44)	South Korea	16 (36 out of 225)	N.A	IRLSSG diagnostic criteria of RLS/WED (2003) 22)
(45)	Iran	14.8 (16 out of 108)	7.5 (32 out of 424)	IRLSSG diagnostic criteria of RLS/WED (2003) 22)
(46)	Finland	20.3 (117 out of 577)	N.A	IRLSSG diagnostic criteria of RLS/WED (2003) 22)
(47)	China	10.7 (28 out of 262)	N.A	IRLSSG diagnostic criteria of RLS/WED (2014) 2)
(48)	Canada	20 (25 out of 123)	5 (6 out of 123)	RLS/WED diagnostic criteria (not specify)
(49)	Brazil	28.4 (25 out of 88)	N.A	IRLSSG diagnostic criteria of RLS/WED (2014) 2)
(50)	Japan	3.4 (15 out of 436)	2.7 (11 out of 401)	IRLSSG diagnostic criteria of RLS/WED (2003) 22)

*RLS/WED, Restless Legs Syndrome/Willis-Ekbom Disease; PD, Parkinson's disease; N.A, not available; IRLSSG, International Restless Legs Syndrome Study Group*.

Several studies supported the hypothesis that RLS/WED and PD may share a common neuropathology (17, 19, 21, 23–25, 32, 34, 38–42, 45, 46). Some surveys reported an increased RLS/WED prevalence in PD patients suggesting a possible relationship between the two disorders, but without supporting any specific pathophysiological bridge (19, 21). For example, Kumar et al. (19) investigated sleep disorders in PD. They found that RLS/WED had a significant higher occurrence in PD patients (14.9%) than in controls (1%). Krishann et al. (21) performed a case-control study showing that RLS/WED prevalence was higher in PD patients (7.9%) than in healthy controls (HC) (0.8%), but the authors did not provide a specific pathophysiological hypothesis. Notably, Nomura et al. (24) found that RLS/WED was more frequent in PD Japanese patients than in Japanese HC, emphasizing an etiological link between RLS/WED and PD beyond the ethnic differences.

Only few studies investigated PD occurrence in a RLS/WED sample. Gao et al. (53) assessed weather RLS/WED can be a preclinical marker of PD by using the Health Professional Follow-up study which evaluated a large sample of men (51,529 males). They employed a sample of RLS/WED patients assessed in according the IRLSSG criteria (22). By considering for analyses only patients who had symptoms at least 5–14 times per month. Among 944 RLS/WED patients 13 also presented PD, while among 22,175 without RLS/WED patients 132 exhibited PD. Thus, the frequency of PD was higher in RLS/WED patients. Furthermore the authors found that patients with more severe RLS/WED symptoms showed higher prevalence of PD. These results were consistent with those of Walters et al. (54), who found that 4.7% of their RLS/WED sample showed also PD in comparison to 1% displayed by general population over 60 years. Dragan et al. (55) collected iRLS/WED patients before the onset of PD. They performed a comparison between RLS/WED with PD group and PD patients without RLS/WED and they found that the former group had a later onset of PD and reduced dyskinesia occurrence. The authors speculated about the possibility that iRLS/WED may reduce the progression of PD.

Other studies evaluated the importance of the duration of the disease and the possible role of the progressive depletion of dopaminergic system. Braga-Neto et al. (23) in a sample of 86 PD patients found that 49.9% of patients exhibited RLS/WED. They highlighted that RLS/WED was more frequent in patients with longer disease duration. Since the occurrence of RLS/WED frequently arose after a mean of 5 years from PD onset, the authors suggested a role of progressive depletion of dopamine system and its occurrence in PD. This hypothesis was supported also by Bhalsing et al. (40) who proposed a possible degeneration of dopaminergic diencephalo-spinal pathway (A11) in the hypothalamus, along with nigrostriatal neurons in PD, that may lead to manifestation of RLS/WED.

Some cross-sectional studies considered RLS/WED as a secondary symptom induced by PD symptomatology and therapy (28, 30, 35, 43, 44, 50). Most of the studies explained the occurrence of RLS/WED as a consequence of dopaminergic therapies. Moreover, the association of RLS/WED with clinical features of PD, especially motor fluctuations have been investigated. Peralta et al. (30) found that 61% of PD patients who met clinical criteria for RLS/WED showed wearing-off. Thus, the authors suggested that the RLS/WED may be part of sensory-motor spectrum of wearing off (RLS/WED-like symptoms). Previously, Fereshtehnejad et al. (45) found similar results. They showed that unpredictability of the off periods was correlated with the higher prevalence of RLS/WED symptoms in patients with PD. Studies conducted in different populations suggest that the antiparkinsonian therapy may explain the occurrence of RLS/WED in PD patients. Lee et al. (28) by a logistic regression showed that the duration of dopaminergic therapy was the factor that better explained the development of RLS/WED in PD patients. Angelini et al. (35) assessed the prevalence of RLS/WED in untreated PD patients excluding secondary forms of RLS/WED and they found no significant differences between patients and controls. Thus, they suggested that RLS/WED occurring in PD patients may be due to dopaminergic therapy.

However, Verbaan et al. (34) studied the prevalence of RLS/WED in 269 PD patients and found a value of 11% that is slightly higher than the frequency reported in general population (56–60) but lower than PD populations of others studies (17, 25, 30), suggesting a possible masking effect of dopaminergic therapy in their sample. They also found that among PD patients RLS/WED severity was positively associated with PD severity, motor fluctuations, depressive symptoms, daytime sleepiness, cognitive problems, autonomic symptoms, and psychotic ones. On the basis of their results, they proposed a non-dopaminergic hypothesis to explain the relationship between PD and RLS/WED. In particular, they emphasized the possible role of adrenergic system in both disorders. The involvement of locus coeruleus and its projections to the central nervous system in both PD and RLS/WED pathologies is supported by some studies (33, 61, 62). In particular, in PD pathology the impairment of serotonergic, cholinergic, and noradrenergic systems in addition to dopaminergic one has been showed (62). Instead the literature regarding this issue in RLS/WED disease is still unclear (61, 33). Thus, the adrenergic hypothesis needs to be more investigated in order to clarify its role in explaining the relationship between PD and RLS/WED.

Other authors explained the higher prevalence of RLS/WED in PD patients with low ferritin levels (17). It is well known that iron has a role in biosynthesis and transmission of dopamine (63). The authors found that 20.8% of their PD patients presented RLS/WED and these patients had lower serum ferritin levels. Thus, they suggested that PD may be a risk factor to develop RLS/WED in combination with low ferritin levels. Interestingly, Fereshtehnejad et al. (45) showed a worse nutritional status associated to RLS/WED in PD patients. They suggested that a worse nutritional status may lead to an iron deficiency in PD patients who exhibited RLS/WED. However, several studies did not show iron deficiency anemia in PD patients with RLS/WED (16, 24, 26, 32, 42, 43).

Shin et al. (43) assessing 151 drug-naïve early-stage PD patients found that 16.5% of PD patients had RLS/WED and presented different characteristics in comparison to RLS/WED of the general population. Indeed, PD patients with RLS/WED tend to perceive symptoms in limb more affected by extrapyramidal symptomatology, while traditional RSL/WED patients have a bilateral involvement. Thus, the authors suggested that RLS/WED in PD patients may have a different underling pathophysiology. Moreover, a significant number of studies reported absence or weak association between the two disorders (16, 20, 26, 27, 36, 37).

Tan et al. (20) reported that none of 125 PD patients recruited met all the clinical IRLSSG criteria for RLS/WED. Loo et al. (26) with a case-control study showed a week association between RLS/WED and PD. Calzetti et al. (27) found that 12.7% of PD and 6.3% of controls suffered from iRLS/WED, but the difference was not statistically significant. Gjertstad et al. (36) evaluated 200 drug-naïve PD patients and 173 healthy controls and found that 15.5% of PD patients and 9.2% of controls had RLS/WED. The difference of prevalence of RLS/WED in the two groups was not statistically significant. The authors also assessed the presence of leg motor restlessness (LMR). LMR was described as an urge to move the legs without met all clinical criteria for RLS/WED. They found that 25% of PD patients and 8.7% HC had a concurrent LMR with a relative risk of 3.1. After the exclusion of the patients with potential confounders the relative risk for LMR was 2.84. These findings supported the notion that RLS/WED and PD may be different entities, but on the other hand opened a debate on whether also LMR and RLS/WED may be considered as such. In relation to this aspect some authors proposed LMR as a bridge between RLS/WED and PD (42). The authors showed that RLS/WED have a similar frequency in patients and controls, but LMR was a more common complaint in PD patients. In addition, no correlation between RLS/WED or LMR and all the possible causes of a secondary RLS/WED evaluated in the study (e.g., neuropathies) has been found.

In conclusion, the literature has not yet been able to give a clear framework of the issue because of the contrasting results. These heterogeneous results may be due to methodological issues. The main problem is the composition of the sample, since different exclusion criteria has been employed among the various studies (e.g., cases of secondary form of RLS/WED like radiculopathies or patients with L-dopa related motor complications). Also the modality of RLS/WED assessment was different across studies (e.g., interviews, neurological evaluation, retrospective use of clinical criteria). Dopaminergic treatment is another remarkable confounding factor. The RLS/WED usually benefits from dopaminergic medication at lower doses than those used for PD treatment (51, 52). Thus, the use of dopaminergic drugs in PD patients may lead to an underestimation of RLS/WED. On the other side, the antiparkinsonian treatment, in particular L-dopa, may produce an increased frequency of sensory-motor disorders in PD (9), giving rise to “mimics” conditions of RLS/WED or augmentation. Notably, considering only studies performed on untreated patients (35, 36, 43) the prevalence range of RLS/WED in PD decreases from 0–50% to 5.5–16.5%.

Moreover, a crucial issue is the time of RLS/WED occurrence in relation to PD onset. Krishnan et al. (21) showed that PD patients with RLS/WED were older than those who did not present the co-occurrence of these diseases. However, other studies found an earlier age at the time of investigation and an earlier onset of PD in patients with RLS/WED (24, 26, 30, 39). Some authors reported a higher prevalence of RLS/WED in female PD patients (25, 26, 34), but others did not find gender differences in PD patients with RLS/WED (21, 40, 43). There is a general agreement in literature considering PD patients with RLS/WED less likely to have a family history of RLS/WED (17, 21, 24, 25, 40, 47). RLS/WED clinical manifestations in PD patients seem to be less severe (24, 25, 46) and RLS/WED symptoms in PD are often transient and irregular (17, 24, 40). However, it must be noted that the studies included treated PD patients, hence the dopaminergic therapy may improve the RLS/WED symptomatology.

## Longitudinal studies

In order to evaluate the causal link between two conditions, longitudinal studies are an essential first step for establishing at least a temporal relationship. However, in literature there are few studies evaluating the incidence of PD in RLS/WED patients, or the appearance of RLS/WED symptomatology in PD patients. Calzetti et al. (64) performed a long-term prospective study to assess the incidence of RLS/WED in newly diagnosed PD patients under dopaminergic therapy. The authors analyzed 106 PD patients with a follow-up ranging from 6 to 96 months. 15 out of 106 (14.15%) patients developed RLS/WED with 3 of them being affected by a secondary form of the disorder: two cases with a chronic polyneuropathy and one case with a bilateral radiculopathy. These prevalence indices are higher in comparison to those reported in a study conducted in general German population in the age ranges of 55–64 and 45–74 years. The median time from starting medication treatment to the development of RLS/WED was 12.5 years with 10 out of 12 patients that developed this condition within 24 months. These findings suggest that dopaminergic medication may be crucial for the development of RLS/WED in PD patients. The same authors reported an updated cumulative incidence and clinical course study in the same cohort of patients after a 3-year follow-up (65). This study confirmed that RLS/WED prevalence is increased in PD patients under treatment in comparison to general population and drug naïve PD patients. The authors demonstrate that clinical course in these patients was prevalently remittent. Accordingly, during an observational period of 12 months after the emergence of RLS/WED, the mean rate of the episodes decreased from 8.9 ± 7.5 in the first 6 months to 3.3 ± 3.2 in the second 6 months. Notably, this time course suggests the absence of augmentation phenomenon in these patients.

More recently, Moccia et al. (66) investigated the presence of RLS/WED patients in a cohort of newly diagnosed PD patients and its incidence after a 4-year follow-up in 109 newly diagnosed PD patients with 10 of them lost during the follow-up. Results showed that RLS/WED is present since the time of PD diagnosis with a prevalence of 4.6% (5 patients out of 109), that rose to 6.5% (7 out of 108), and 16.3% (16 out of 99) after 2 and 4 years. Incidence rate was 5.7% at 2 years and 10.2% at 4 years, with a cumulative incidence of 6.8%. However, no significant association was found between dopaminergic therapy and RLS/WED. Interestingly, this study investigated also dopamine transporter by means of single photon emission computed tomography (FP-CIT SPECT). Findings demonstrate that PD patients with RLS/WED showed a preserved nigrostriatal dopaminergic pathway in comparison to patients without RLS/WED. This result seems to suggest the involvement of neurotransmitters diverse from dopamine.

Up to now only two longitudinal studies evaluated the presence of RLS/WED as an early manifestation or risk factor of PD. In 2014, Wong and Li (67) performed a prospective longitudinal study assessing 22,999 health professional men aged 40–75 without PD, diabetes, arthritis and common mimics of RLS/WED with an 8-year follow-up. At baseline evaluation 931 subjects affected by RLS/WED were identified. Among these, 7 out of 8 incident PD cases were observed during the first 4 years of follow-up. Furthermore, a significant risk for developing PD in subjects affected by a severe form of RLS/WED (RLS/WED symptoms 15+ times/month) in comparison to subjects without RLS/WED was found during the same time period, but not in the full 8-year follow-up. According to the authors' interpretation of the results, these data suggest that RLS/WED might be an early manifestation of PD rather than a risk factors, since a longer follow-up period was not associated with an increased risk of PD development. Therefore, they speculate a different pathogenesis for these two disorders.

More recently, Szatmari et al. (68) evaluated the association of RLS/WED with the development of incident PD in a large cohort of US veterans. Out of 3.5 million of US veterans, 58,475 had a prevalent RLS/WED. After a mean follow-up of 8.1 years, 68 incident PD were identified in the no-RLS/WED group in comparison to 185 PD in the RLS/WED group. Therefore, a two-fold increased risk for PD was found in RLS/WED patients. The authors argued that since the uncertainty regarding the pathophysiological mechanism of RLS/WED and the low incidence of PD in this condition, it is very difficult to speculate regarding a common ethiopathogenesis between the two disorders.

The increased incidence of RLS/WED in PD patients is supported by all three longitudinal studies (64–66), however the possible influence of dopaminergic therapy in inducing RLS/WED is reported in two out of three (64, 65). Furthermore, neuroimaging showed a preserved dopaminergic pathway in PD+RLS/WED in comparison to PD alone. On the other hand, the two studies investigating the development of PD in these patients seem to indicate that RLS/WED might be an early manifestation rather than a risk factor of the neurodegenerative disease.

Alongside with the paucity of longitudinal studies investigating this association, several limitations have to be taken into account interpreting these results. When considering studies investigating the insurgence of RLS/WED in PD patients, the most striking weakness common to all three longitudinal studies (64–66) was the lack of a control group. Furthermore, a possible underestimation of the disorder due to the presence of dopaminergic treatment, that likely permit to identify only those patients affected by a severe form or those who did not respond to this therapy, might be considered. In the two studies examining the incidence of PD in RLS/WED patients (67, 68) the main concern regards the assessment of RLS/WED. In one study (67) the presence of the disorder was assessed throughout a questionnaire, whereas in the other (68) it was retrospectively extracted from a database through the codes of the International Classification of Diseases (Ninth Revision). For these methodological issues, results should be cautiously interpreted.

## Directing glance on dopaminergic system physiology: can the dopamine be a reliable bridge between RLS/WED and PD?

Dopamine (DA) is the most common catecholamine in the central nervous system that can modulate different functions, like movement, cognition, reward and motivation (69, 70). DA derived from the conversion of 2,3-dihydroxyphenylalanine (DOPA) by the enzyme DOPA decarboxylase (DDC). Tyrosine hydroxylase (TH) is the enzyme responsible for converting the amino acid tyrosine to DOPA, monitoring the DA amount.

It is known that there are three groups of dopaminergic cells that give rise to three different axonal pathways with different functions: nigrostriatal, mesocorticolimbic, and tuberoinfundibular system. The latter is the smallest in terms of brain DA content and controls the pituitary system. Nigrostriatal DA pathway controls voluntary movement, and dysfunction in this pathway has been implicated in movement disorder like PD. Mesocorticolimbic systems DA modulate various cognitive/emotive functions, and their degeneration may lead to some psychiatric disorders. Several studies have pointed out that mesocorticolimbic system can also modulate thalamocortical arousal state (71–73). Studies from the effect of psychomotor stimulant with a molecular structure similar to DA, like amphetamine, (74), has demonstrated that endogenous DA is involved in promoting wakefulness (75, 76).

It is also known that DA release has a circadian fluctuation, and his effects on the DA receptors are different during the day and the night, with a high-affinity for D2-like receptor during the night whereas the effect on D1 receptor can overwhelm the actions of D2-like receptors during the day (15). The sleep/wake effects of exogenous dopaminomimetics, drugs typically used in diseases such as PD but also RLS/WED, are dose and receptor dependent. Sleep is promoted by low dopaminomimetic dose via D2-like receptors (77, 78), whereas higher dose enhances wakefulness via D1-like postsynaptic receptors (79–81).

Take into consideration his contribution to sleep-wake state in addition to other waking behaviors like movement, DA has been considered the “bridge” that underlying PD and RLS/WED (15).

Depletion of DA in basal ganglia as pathophysiology basis of PD is known from 1960s. DA deficiency in the nigriostriatal pathway causes denervation hypersensitivity of D1 and D2 receptors, highly concentrated in the dorsal striatum (82). On the contrary, D3 receptors, more abundant in the mesolimbic pathway (83), are decrease by 40 to 45 percent in PD patients (84) and this can explain the hypersensitivity of D2 nigrostriatal receptors observed in PD. The pathogenesis of neuronal cell degeneration in the basal ganglia is still debated. Numerous theories have been suggested (85).

The dopaminergic pathology has been proposed also among the pathophysiological mechanisms of RLS/WED, as confirmed by the efficacy of the therapy with L-Dopa and DA agonists in the clinical and polysomnographic improvement of patients with RLS/WED (86).

Unlike PD, in RLS/WED anatomopathological studies (87, 88) and some Cerebral Spinal Fluid (CSF) studies (89–91) have failed to provide a consistent pattern indicating a DA deficit.

More recently, a human postmortem study had demonstrated significant decrease in D2 receptors in the putamen and a significant increase in TH in the SN, showing no differences for D1 receptors, DA transporter or vesicular monoamine transporter (VMAT), as in animal models of iron depletion, confirming a clear DA pathology in RLS/WED patients with an increased DA production and DA receptors downregulation, secondary to a primary iron insufficiency (92).

The hypothesis of a hyperdopaminergic state is supported by another study that showed increased levels of the DA metabolite 3-ortho-methyldopa (3-OMD) in CSF of patients with RLS/WED compared to controls (93). In particular, 3-OMD levels are increased during the day but reduced at night, suggesting that in RLS/WED patients there may be a relative DA deficiency during the night on a hyperdopaminergic state on the background (91). Hyperdopaminergic state leads to a downregulation of DA receptors, but due to the circadian profile of DA activity, there is a relative hypofunctioning in the evening and during the night, explaining the relief of RLS/WED symptoms after supplying additional DA with dopaminomimetic drugs (94). Clinical phenomenon of augmentation confirms this theory. DA-based medicines can cause further downregulation or desensitization of DA receptors, increasing the DA requirements while DA deficiency during the night becomes more severe and tends to occur for longer periods with a worsening of RLS/WED symptoms (95).

Interestingly all CSF studies have consistently shown iron insufficiency in RLS/WED (96–99), and autopsy analysis demonstrated alteration iron regulatory protein 1 in neuromelanin cells indicating iron deficiency (100).

Imaging studies in RLS/WED patients have tried to demonstrate the physiopathology of this disease, but with some discordances (101). Regarding the dopaminergic hypothesis Positron Emission Tomography (PET) and Single-photon emission computed tomography (SPECT) studies support a dysfunction in both nigrostriatal and mesolimbic pathways (102–107). D2 receptors and DA transporters in the striatum appear decreased, and these findings are compatible with an increase in synaptic DA (102–105, 107–109).

Using Magnetic Resonance Imaging (MRI) iron-sensitive sequences, numerous evidence has shown iron deficiency in RLS/WED patients (110–116) supporting the iron-dopamine bridge hypothesis (117).

Regarding the principal clinical manifestation of RLS/WED, functional Magnetic Resonance Imaging (fMRI) studies have demonstrated connectivity changes in cerebral areas implicated in the limbic/nociceptive network and the sensorimotor network (118–123). Also, SPECT studies have demonstrated an involvement of the limbic structures, as medial thalamus and anterior cingulate cortex (124, 125).

Latest evidence supports the notion that RLS/WED represents a complex network disorder, with the crucial node localized in the thalamus, which appears to have dopaminergic dysfunction (126), lower iron content (114, 115), and changes in activation and functional connectivity (112, 118, 120).

## Iron and its relation to the dopamine system

Iron is an important cofactor in several DA metabolisms and can also produce neurotoxic species.

Usually iron accumulates in the normal aging brain, in particular in the putamen, globus pallidus, red nucleus, and substantia nigra (SN) (127). Elemental iron plays a critical role in oxidative metabolism and it also serves as a cofactor in the synthesis of neurotransmitters (128).

In PD, neurodegeneration occurs mainly in SNc (129), while other iron-rich areas remain unaffected. In early stages of the disease the identification through the use of transcranial ultrasonography of a hyperechogenicity of the SNc (130) correlates positively with the increase of iron and ferritin evaluated in post-mortem analysis (131), allowing an early identification of patients at risk for PD (132).

The increase in neuronal iron may be secondary to an increase in influx, facilitated by transferrin receptor-2/divalent metal transporter-1 endocytosis or the diffusion of ferric citrate (133), an increase in efflux, due to alteration of the activity of ceruloplasmin, or a dysregulation of iron homeostasis, mediated mainly by the iron storage protein ferritin (134).

Some studies have shown reduced ferritin concentrations in the SN from Parkinson's disease brain, suggesting an alteration of this storage mechanism and a consequent increase in the level of free and potentially harmful iron (135).

Also, neuromelanin, a final product of DA, can be implicated in the dysregulation of iron metabolism (136). Quantitative imaging showed in PD patients a significant elevation in iron levels in SNc neuromelanin-positive cells compared with locus coeruleus (137). It is not clear if the association of iron with neuromelanin can play a role in the degeneration of SN cells, but it is hypothesized that when this pigment becomes saturated, an excess of iron can be released into the cytoplasm (138).

DA metabolism through oxidation by iron and oxygen can form o-quinones and 6-hydroxydopamine (6-OHDA) (139). These quinones can form neurotoxic intermediates in iron-facilitated reactions, resulting in alteration of cell membrane integrity and, eventually, cell death (140). 6-OHDA induces mitochondrial dysfunction (141) and can liberate iron from ferritin (142) that in high concentrations overwhelms compensatory antioxidant mechanisms (143) and facilitates the production of further neurotoxic species.

In PD patients iron is increased by about 50 percent in SN compared to controls (144), and this finding supports the hypothesis that abnormal iron metabolism plays a pathologic role in the development of PD (145, 146).

On the other hand, there is some evidence that links RLS/WED to iron deficiency states. High prevalence of RLS/WED was found in specific condition implicating a reduction in the availability of iron such as pregnancy, iron deficiency anemia or renal pathologies (147–149). However, iron levels in blood sample of most RLS/WED patients are normal (117), suggesting that a state of low iron in the brain could be implicated in the RLS/WED pathophysiology.

MRI study have demonstrated a significant low concentration in specific brain regions as SN, and these decreases were correlated to RLS/WED symptom severity (110). Other imaging studies found iron decrease in other brain regions, like thalamus, caudate, putamen, and white matter (114).

Immunohistochemistry postmortem RLS/WED brain samples have shown a significant reduction of iron and ferritin in the SN (150), as subsequently confirmed in CSF analysis of RLS/WED patients (97, 99, 151).

Iron deficiency may lead to an increase of TH in the basal ganglia (92) and elevated extracellular DA levels (152). Also, DA receptor density may be modified by iron deficiency, with a reduction in caudate and putamen D1 and D2 receptors (87).

Several studies have implicated both the dopaminergic system and the iron in PD and RLS/WED, thus suggesting a common physiopathological basis, however the data are inconsistent with this theory, showing in particular a depletion of DA in PD and a hyperdopaminergic state in RLS/WED.

## Conclusion

The relationship between RLS/WED and PD has been largely investigated by cross-sectional and longitudinal studies. Among the different pathophysiological hypotheses emerged by cross-sectional studies two of them are confirmed also by longitudinal investigations. In particular, a twofold interpretation regarding the association between these two conditions has to be taken into account: dopaminergic therapy may have a crucial role in the development of RLS/WED in PD patients (28, 35, 64, 65) and on the other hand RLS/WED can be conceived as an early manifestation of PD rather than a risk factor (42, 64, 65). Therefore, it is plausible that these two hypotheses differ in etiopathogenetic mechanisms. However, the literature regarding the pathophysiology of the two diseases showed different results struggling to give a clear message on the possible bridge between RLS/WED and PD. Despite numerous studies showing a higher prevalence of RLS/WED in PD patients and several findings related to dopaminergic and iron alterations in both disorders, up to now it is difficult to find a point of agreement between studies.

Conflicting results may be explained by methodological and theoretical issues. Confounding variables, such as therapy, mimic conditions, time course, symptoms' features, diagnostic criteria, and disease duration should be seriously considered and controlled when investigating the incidence or the prevalence of RLS/WED in PD. Furthermore, whereas there is a certain degree of accordance regarding PD pathophysiology, physiological mechanisms underlying RLS/WED are poorly understood and still matter of debate.

The presence of RLS/WED in PD patients may be partially covered by the presence of dopaminergic therapy or represents a minor sensorimotor complaint among those already present in PD. Furthermore, in both published literature and clinical experience, the long-term observation of RLS/WED patients does not provide evidence regarding a frequent incidence of PD.

In order to better understand this relationship, a greater number of systematic and strongly controlled longitudinal studies are needed. At the same time, it is necessary to improve the knowledge on the pathophysiology of RLS/WED in order to fill the gap regarding putative common etiopathogenetic mechanisms shared with PD.

## Author contributions

Conception of the work, literature search and interpretation: LF-S, GC, FC and AG. Drafting and revising the work critically for content: LF-S, GC, FC and AG. Final approval of the version to be published: LF-S, GC, FC and AG.

### Conflict of interest statement

The authors declare that the research was conducted in the absence of any commercial or financial relationships that could be construed as a potential conflict of interest.
